# Immunomodulatory Properties of *Coriolus versicolor*: The Role of Polysaccharopeptide

**DOI:** 10.3389/fimmu.2017.01087

**Published:** 2017-09-06

**Authors:** Mohammad H. Saleh, Iran Rashedi, Armand Keating

**Affiliations:** ^1^Institute of Biomaterials and Biomedical Engineering, University of Toronto, Toronto, ON, Canada; ^2^Cell Therapy Program, Princess Margaret Cancer Centre, Krembil Research Institute, University Health Network, Toronto, ON, Canada

**Keywords:** immunomodulation, mushroom, *Coriolus versicolor*, Yun zhi, polysaccharide peptide, polysaccharopeptide

## Abstract

Traditional uses of herbal medicine have depended mostly on anecdotal evidence for much of history. The increasing application of scientific rigor to the study some of these traditional therapies in recent years has revealed potent bioactivity, notably demonstrated by the 2015 Nobel Prize for the discovery of an antimalarial compound from traditional Chinese herbs. Given the recent successes of immunotherapy and checkpoint blockade, there is a renewed interest in identifying new drugs with immunomodulatory effects. As an estimated 45–60% of cancer patients worldwide are reported to use complementary alternative medicine alongside traditional therapy, this review will highlight the literature on the immunomodulatory effects of one of these compounds. We report on the induction of a largely pro-inflammatory cytokine profile by the polysaccharopeptide (PSP) isolated from the *Coriolus versicolor* (Yun zhi) mushroom, as well as its effects on various immune subsets, and the clinical data that have led to its widespread adoption as an adjunct cancer therapeutic in many Eastern cultures. Particular focus is given to the potential mechanisms underlying the bioactivity of PSP and reports of its ability to promote antitumor immunity by helping overcome tolerogenic tumor microenvironments.

## Introduction

Herbal remedies have long played a role in human well-being and depend mostly on anecdotal and historical reports to support their use. Over the last three decades, the application of scientific methodology has partly elucidated the underlying mechanisms of some herbal treatments. Indeed, many chemotherapeutic agents currently in use, including topotecan (1), etoposide, teniposide (1, 2), docetaxel, and paclitaxel (3), are derived from herbal origins. With the recent and growing interest in immunotherapy, some investigators have turned to natural products in search of immunomodulatory compounds (4).

A particularly interesting source of immunomodulatory agents is the *Coriolus versicolor* (CV) mushroom, also referred to as *Yun zhi* (China), Kawaratake (Japan), Turkey tail (North America), or any of *Agaricus/Boletus/Polyporus/Poria/Trametes versicolor* plants. Ancient Chinese formulations of CV have long been believed to generally promote health, strength, and longevity. Laboratory studies suggest it may have antimicrobial, antiviral, and antitumor properties. Products using CV extracts are currently approved as adjunct therapy in China and Japan for cancer patients already receiving chemotherapy or radiotherapy.

The bioactive components of CV extracts include two polysaccharopeptides (PSPs) derived from two different strains of CV: COV-1 (PSP) most commonly used in China and CM101 (polysaccharide krestin, PSK) used in Japan. Both molecules are about 100 kDa with respective polysaccharide-to-peptide balance of 90–10% in PSP, and 60–40% in PSK. The carbohydrate moieties of each compound consist of mannose, xylose, galactose, in addition to fructose in PSP or arabinose and rhamnose in PSK. PSP is typically isolated by boiling COV-1 mycelia or fruiting bodies in water, followed by precipitation in ethanol.

Numerous studies *in vitro, in vivo*, and some clinical trials have reported immunopotentiation by *Yun zhi*—leading to its adoption as an adjunct therapy for cancer in many Eastern countries. This review aims to summarize the English literature on the immunomodulatory effects of CV extracts, with a particular focus on PSP. Reports of its effects on cytokine release, and especially on its potential to activate natural killer (NK) cells, are followed by examining the activity of PSP on inflammatory immune subsets and a brief overview of relevant clinical studies.

*Coriolus versicolor* extracts are used as adjunct therapy for cancer in many Eastern countries.

## PSP Induces a Predominantly Pro-Inflammatory Cytokine Profile

The best reported immunomodulatory effect of PSP is its induction of predominantly pro-inflammatory cytokines. *Yun zhi* has a potent effect on *in vivo* and *in vitro* expression of tumor necrosis factor (TNF)-α, commonly of interest for its ability to induce apoptosis and its potent tumoricidal activity (5). Primary mouse peritoneal macrophages treated with PSP *in vitro* showed increased TNF-α release comparable to levels achieved by lipopolysaccharide (LPS) stimulation (6). When human peripheral blood mononuclear cells (PBMCs) from healthy donors were incubated in PSP for 18 h, either in the presence or absence of phytohemagglutinin (PHA, a T cell mitogen), there was a more than 3.5-fold increase in TNF-α secretion (7). PBMCs from breast cancer patients also exhibited increased *TNF-α* expression and protein production in response to PSP, an effect not abrogated by blockade of toll-like receptor 4 (TLR4), suggesting that PSP is independent of TLR4 activation (8). *In vivo*, peritoneal macrophages from healthy mice injected intraperitoneally (i.p.) with PSP produced significantly higher amounts of TNF-α compared with saline controls (6). A similar increase in TNF levels was observed when mice with subcutaneous (s.c.) tumors derived from the herpes virus Type 2 transformation of a murine fibroblastic origin (the H238 line) received PSP s.c. for 11 days. Tumor specimens from mice treated with PSP alone had higher expression of TNF-α than untreated controls (9). Administration of PSP i.p. also resulted in a significant increase in serum TNF levels in healthy rats (10, 11).

The ability of PSP to induce cytokines associated with TNF-α was also demonstrated through its induction of IL-12, a T helper 1 (Th1)-related cytokine capable of enhancing NK and CD8^+^ T cell cytotoxic activities and their expression of TNF-α. The incubation of murine splenic lymphocytes with CV extract for 48 or 72 h resulted in a threefold increase in IL-12 production compared to control samples (12). Similar effects were demonstrated in PBMCs from breast cancer patients, showing an increase in protein production of IL-12 after treatment with PSP and PHA (8). Moreover, IL-12 is a known inducer of interferon (IFN)-γ, a potent immunostimulatory cytokine, and PSP consistently leads to IFN-γ expression. PBMCs from healthy donors and breast cancer patients showed increased production of IFN-γ when treated with PSP and PHA, respectively, for 24, 15, or 72 h (7). Similarly, 24-h incubation in CV extract resulted in increased production of IFN-γ by murine splenic lymphocytes. Incubation of these cells was also able to increase IL-2 (almost eightfold) and IL-18 (about twofold), both of which are Th1-related cytokines able to induce IFN-γ production by NK and T cells (12).

Polysaccharopeptide can also induce the pleiotropic cytokine, interleukin-1β (IL-1β). Classically considered a pro-inflammatory signal, it is typically elicited in response to TLR signaling and can serve to enhance lymphocyte proliferation and differentiation (13). Primary mouse peritoneal macrophages treated with PSP *in vitro* induced IL-1β in a time- and dose-dependent manner up to levels comparable to induction by LPS (6). When these healthy mice were injected i.p. with PSP, their peritoneal macrophages were capable of producing significantly higher amounts of IL-1β compared with saline controls (6). Another closely related cytokine affected by PSP is IL-1α. When human PBMCs were cultured with PSP for 24 h, IL-1α production increased threefold (14). While also pleiotropic in its effects, IL-1α can serve to costimulate CD8^+^ T cells and enhance antigen presentation by tumor cells (15). In addition, the increased release of granulocyte–macrophage colony-stimulating factor (GM-CSF) and granulocyte colony-stimulating factor (G-CSF), stimulators of hematopoiesis that tend to be pro-inflammatory but also have pleiotropic effects, by PBMCs from healthy donors were observed upon culture with PSP and PHA (14). Similar effects were observed when healthy mice injected with PSP led to increased expression of macrophage colony-stimulating factor (*M-CSF*) (16), a related multifunctional growth factor.

Extracts of CV have in addition been shown to affect the expression of other pleiotropic cytokines, including transforming growth factor (TGF)-β, which has pro-inflammatory effects on monocytes and Th17 cells, and anti-inflammatory effects on B cells, T regulatory cells (Tregs), and activated macrophages (17, 18). In mice carrying s.c. H238 tumors treated s.c. with PSP alone for 11 days, tumor specimens had a slight decrease in TGF-β staining (compared with untreated controls), which was significantly augmented by IL-2 cotreatment (9). PSP also affected levels of IL-6, which has pro-inflammatory activity through its enhancement of B cell antibody production and B helper activity, inhibition of Treg differentiation, and induction of acute phase proteins (19). IL-6 also has anti-inflammatory activity *via* inhibition of TNF-α and IL-1 (20) and plays a role in the generation of myeloid-derived suppressor cells (MDSCs) (21). Healthy rats with prolonged LPS-induced fever due to PSP pre-treatment (i.p.) exhibited elevated blood IL-6 levels and this prolongation was abrogated by anti-IL-6 antibody treatment (14). Similar results showed that PSP treatment of the acute myeloid leukemia line, HL-60 resulted in a dose-dependent increase of IL-6 release (22), while 24-h treatment with PSP and PHA of human PBMCs from breast cancer patients also showed increased IL-6 expression (8). Similar results were obtained with PBMCs from healthy donors (14).

In addition to directly affecting cytokine release by immune cells, CV extracts increase the sensitivity of these cells to other stimuli and exert a synergistic effect with other factors. For example, peritoneal macrophages isolated from healthy mice orally administered PSP showed increased TNF-α production in response to LPS stimulation (23). Culturing human liver carcinoma cells, HepG2, with a non-toxic dose of PSP increased their susceptibility to cyclophosphamide cytotoxicity in a synergistic manner (24). Testing PSP on the herpes-transformed H238 murine line *in vitro* decreased their DNA synthesis in a manner that synergized with the supplementation of IL-2 (23). Taken together, these studies implicate a stimulatory effect of CV extracts on the immune system.

PSP induces production of many pleiotropic cytokines with predominantly a pro-inflammatory profile, which act locally and systemically.

Polysaccharopeptide appears to protect against the adverse effects of radiation. Intragingival administration of CV extract for 10 days reversed decreases in spleen weight and splenocyte DNA synthesis after healthy mice received a single dose of 1 Gy whole-body irradiation (25). Interestingly, in a s.c. tumor model, mice that received radiation alone had the lowest tumor growth compared with groups that received PSP with or without radiation. This may be due to PSP’s ability to induce scavenging of oxygen radicals (thereby weakening radiation efficacy) and/or through enhanced lymphoid infiltration of tumors in the presence of PSP. In support of the latter, PSP-treated animals (particularly those not subjected to any radiation) showed increases in phagocytic, NK, T, and B cell counts in their blood and spleens (26). This systemic increase in immune activation could have led to augmented tumor infiltration (and thus apparently larger tumor volumes), but this cannot be definitively concluded as the immune subsets within the tumor microenvironment were not reported in that study.

### Antitumor Effects

The most commonly used model to study the tumoricidal effects of PSP is *in vitro* culture of human leukemia HL-60 cells, which have demonstrated reduced proliferation by disruption of their cell cycle (27–30), induction of apoptosis (28, 29, 31), and sensitization to various chemotherapeutics such as camptothecin (30), doxorubicin, and etoposide (27). These effects correlate with decreases in anti-apoptotic proteins bcl-2 and survivin along with increases in bax and cytochrome *c* (29), as well as decreases in various phosphatase and kinase genes (28), and the activation of caspase-3, -8, and -9 (31). Treating human PBMCs with PSP using conditioned media to grow HL-60 or U937 showed significant tumoricidal activity that could be inhibited by antibody blockade of either of TNF-α or IFN-γ (32). TNF-α induction was also observed by administration of PSP *in vivo*. However, despite showing pro-inflammatory effects consistent with other studies, PSP can exhibit no direct cytotoxic effects on murine lines of hepatoma (33), sarcoma, melanoma (23), breast cancer (34), or human lines of placental choriocarcinoma (23, 33). *In vitro* assays of cell migration on 4T1 murine breast cancer cells treated with PSP revealed inhibition of migration in a time- and dose-dependent manner and significant reduction of matrix metalloprotease, MMP-9 production. This was reflected *in vivo* when mice injected with 4T1 cells showed decreased growth of lung but not liver metastases in response to PSP treatment (34). While some of the differences in cell types may explain inconsistency of the direct cytotoxicity of PSP, there are contradictory reports showing apoptosis of even the same cell line, HL-60 (28–31). These discrepancies may be due to variation in the preparation and extraction methods of PSP. Indeed, PSP’s ability to inhibit the growth of another human leukemia cell line, Molt-4, significantly depended on the fermentation duration of CV prior to its harvest (7), demonstrating the need for additional rigorous studies to standardize and optimize the yield and activity of PSP from CV.

The antitumor effects of PSP should be carefully studied in the context of complex networks of cancer-related immune response and tumor microenvironment. In a study, mice were pretreated (s.c.) with PSP for 5 days prior to, or started on PSP on the same day as, s.c. tumor cell implantation, and then followed by radiation a week later (26). Both groups showed decreased tumor growth compared to non-treated control animals, with the pretreated group showing slower growth. This may partially be mediated by increases in local or systemic IL-1β with PSP pretreatment (13), thus effectively enhancing lymphocyte proliferation prior to tumor implantation. Indeed, mice that received PSP alone on the day of tumor implantation showed only modest effects compared with untreated controls, suggesting that PSP alone has insufficient tumoricidal effects in this model. Notably, the group that received PSP on the same day as radiation (a week after tumor implantation) showed no effect compared to untreated controls, highlighting that to appropriately sensitize tumors to radiation therapy, PSP may need to be administered well in advance of radiation in what may be a time-dependent relationship. Similarly, the increase in IL-1β production by PSP in the tumor microenvironment may have counterproductive effects on tumor growth since expression of IL-1β in the tumor milieu promotes tumor invasiveness, angiogenesis, and tumorigenesis (15). IL-1 can, in some cases, increase tumor immunogenicity and decrease invasiveness (15). The risk of deleterious effects holds even in the context of significant increases in systemic IL-1β, since it can promote the expansion of MDSCs (15). Likewise, PSP-induced increase of IL-6 expression, in the context of enhanced IL-1β and GM-CSF expression, may lead to increased levels of MDSCs or Tregs at the tumor microenvironment. It is important to note that reports of IL-6 induction have shown systemic increases, while the only *in vivo* studies of IL-1β induction have shown its increase in a localized setting (i.p.) (6). Because PSP is usually administered orally in clinical settings, it is important to conduct more clinically relevant experiments to study the effects of its oral administration on systemic levels of IL-1β in animal models. It may, in fact, be that the typical route of delivery of PSP provides beneficial local pro-inflammatory while averting the potential detrimental effects of IL-1β expression systemically or at the tumor site.

In addition, while generation of MDSCs at the tumor site depends on TGF-β, PSP seems to moderately decrease its expression (9). On the other hand, the increase of GM-CSF expression, combined with an IL-6 increase, may lead to MDSC formation (21). Thus, while some of the induced cytokines may lead to enhanced tumor progression or immune suppression, more careful examination of PSP’s effects on immune subsets (particularly MDSCs and Tregs) in the tumor environment is necessary for a conclusive understanding of PSP’s immunomodulatory effects and how to best put them to use. Much of the literature has depended on *in vitro* or healthy animal *in vivo* experiments and more studies using tumor models that enable careful examination of the effects of PSP on cytokine induction. For example, along with staining for MDSCs in tumor samples after PSP treatment, the administration of PSP could be tested alongside MDSC depletion by anti-Gr1 or anti-Ly6G antibodies to test whether a combination therapy further slows tumor progression.

Rigorous studies are needed to standardize preparation methods, optimize kinetics, biodistribution, and activity of PSP to better establish specific immunomodulatory effects.

## Effect of PSP on Immune Cell Populations

Polysaccharopeptide has a wide range of mostly stimulatory effects on other immune cell types. The proliferation of cultured splenocytes was significantly augmented by incubation with CV extract *in vitro* (12). Human PBMCs incubated in PSP and PHA showed a time-dependent increase in proliferative responses (7), while non-stimulated human lymphocytes treated with PSP showed a dose-dependent enhancement of proliferative activity (35). A 48-h culture with PSP decreased Fas receptor expression of non-stimulated lymphocytes and synergized with cyclosporine for a similar effect on PHA-stimulated cells, hinting at a protective role of PSP against extrinsic death signals (35). PSP can also affect monocytes and macrophages. Healthy human PBMCs treated for 48 h showed an increase in the number of CD14^+^CD16^−^MHCII^+^ monocytes (36), while several studies suggest that PSP increases the phagocytic activity of macrophages in culture and *in vivo* (37). The *in vitro* treatment of purified murine splenic B cells with CV extract showed a strong proliferative response, which can be inhibited by BCR blocking antibody, implicating its role in CV-mediated B cell activation (38).

Similar proliferation and activation responses were observed in various animal studies as well. After oral administration of PSP to healthy mice for 2 weeks, isolated peritoneal macrophages showed increased production of reactive nitrogen intermediates and superoxide anions in response to *in vitro* stimulation (23), while macrophages and T cells from healthy mice force-fed CV extract for a week showed enhanced nitrite production and proliferative mitogenic response, respectively (33). Lymphocytes isolated from rats administered PSP orally for 2 weeks showed enhanced lymphocyte proliferation and rescue from the negative effect of cyclophosphamide chemotherapy, while NK cells from these healthy animals showed a comparable trend in cytolytic function (39). These results are noteworthy, given the typical route of administration for PSP in a clinical setting is oral. In addition, the treatment of healthy nude mice (i.p.) with CV extract for 2 weeks resulted in an increased white blood and neutrophil count (37). Mice carrying s.c. H238 tumors injected s.c. with PSP exhibited an increase in lymphocyte numbers and, when combined with IL-2, in splenocyte stimulation by PHA. PSP also slowed the tumor progression of these mice, although not as effectively as IL-2 alone (9).

Some of these enhanced proliferative effects on leukocytes are likely due to the pro-inflammatory cytokines induced by PSP, but that may not be the sole factor in play. When murine peritoneal macrophages were isolated after a 5-min i.p. treatment with PSP, they exhibited 1.8-fold heightened release of prostaglandin E_2_ (PGE_2_) (6). Prostaglandins are a group of hormone-like lipid compounds with multiple effects, including the regulation of inflammation (40). In particular, PGE_2_ has previously been shown to stimulate Th1 differentiation (41, 42), alter dendritic cell (DC) migration and costimulatory molecule expression (41, 43), and enhance TNF-α production by NK cells (44). The combination of PGE_2_ with other previously reported cytokines induced by PSP (specifically TNF-α, IL-1β, and IL-6) can enhance the migratory and immunostimulatory capacity of DCs in culture (45). These cells play a crucial role in contact-dependent activation of NK cytolytic activity and are often activated by NK-derived IFN-γ and TNF-α (46). While PGE_2_ has also been shown to have anti-inflammatory effects through the Th2 axis, many have argued that its activity is pleiotropic and largely context dependent. Again, it is important to understand that this increased PGE_2_ expression may inadvertently enhance MDSC maturation (21).

PSP has stimulatory effects on many immune cell types, enhancing their proliferation and cytokine release.

### Effects on Adaptive and Innate Immune Responses

*Coriolus versicolor*-derived compounds have a marked effect on humoral immunity. Murine splenocytes enriched for B cell populations and treated with CV extract for 6 days in culture revealed a potent ability to induce IgM production and, when combined with exogenous IL-4, IgG1 secretion (38). The systemic administration of CV extract for 2 weeks to healthy nude mice gave an almost twofold increase in serum IgG levels compared with saline-treated controls (37). Similarly, lymphocytes isolated from healthy rats fed PSP for 2 weeks showed increased IgG production compared to saline control rats, which was only slightly hampered by the addition of cyclophosphamide (39). Administration of PSP in combination with acacia gum by oral gavage to healthy mice for just 4 days showed a significantly increased IgG production when compared to those receiving acacia gum alone (47). Taken together, these studies suggest that PSP may function as an adjuvant, mediating humoral responses *via* T cell-dependent increases in B cell activity and the generation of a non-specific polyclonal antibody response (37).

**Figure 1:**
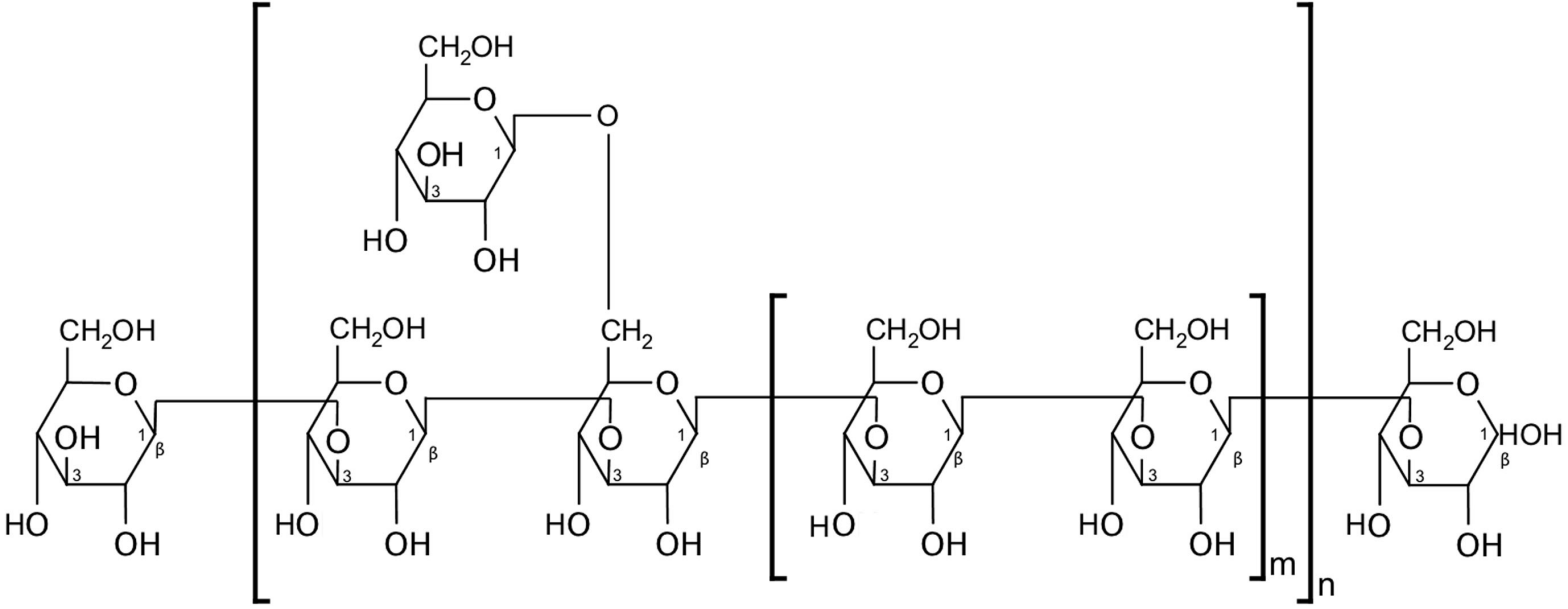
Partial structure of polysaccharopeptide (PSP). PSP’s polysaccharide moiety contains a β-(1→3)-d-glucan. Figure replicated from Wan et al. (49).

A growing body of evidence suggests the involvement of CV ingredients in the activation of various pattern-recognition receptors (PRRs)—a crucial step in initiating the innate immune response upon encounter with a pathogen-associated molecular pattern (PAMP). β-glucan polysaccharides, one of which is the carbohydrate moiety of PSP [a β-(1→3)-d-glucan that branches at positions 4′ and 6′, Figure 1] (48, 49), can act as PAMPs, stimulating an array of murine and human PRRs. For example, the complement receptor 3 (CR3) is a PRR highly expressed on monocyte and NK cell surfaces and is associated with extravasation of these cells toward pathogens, along with initiating phagocytosis and degranulation. CR3 has two binding sites and is thus activated by pathogens or immune complexes containing β-(1→3)-glucans and an opsonin (48, 50). Another PRR that is primarily stimulated by β-glucans is Dectin-1, which is expressed by monocytes, macrophages, and to a lesser extent, DCs and some T cells (48, 51). Indeed, the β-glucans extracted from CV were shown to increase phagocytic activity and the release of TNF-α and nitric oxide by peritoneal macrophages after 4 days in culture—effects that were abrogated upon inhibition of Dectin-1 signaling (52). Moreover, β-glucans were also shown to interact with TLRs. For example, the increase of TNF-α but not IL-6 in some cases *in vivo*, after administration of PSP alone, suggests that PSP may act *via* the TLR-4/p38 MAPK pathway (14). This is supported by data showing that splenocytes from C3H/HeJ mice (i.e., carrying an inactivating mutation in TLR-4) treated with CV extract in culture exhibited lower proliferation than their normal counterpart, with cells showing a distinct time-dependent increase in p38 MAPK phosphorylation upon CV treatment (38). Healthy human PBMCs also treated with PSP and PHA for 24 h showed upregulated expression of TLR-associated genes, including *TLR4, TLR5, TLR6, TLR7*, and *LY64* (14), while multiple genes, kinase phosphorylation levels, and proteins in the TLR4 pathway were significantly upregulated by PSP and PHA treatment of PBMCs from breast cancer patients (8). However, as mentioned earlier, anti-TLR-4 antibody blockade did not abrogate the beneficial effects of PSP on cytokine production (8), pointing to the possible involvement of multiple signaling pathways in response to PSP. In addition, using neu transgenic mice, PSK was also shown to mediate DC and T cell activation *via* TLR-2 signaling (53). Further examination of immediate molecular responses by lymphocytes, and tumor cells, to PSP will illuminate the underlying mechanisms involved in its activity and may enable the maximization of PSP’s beneficial effects.

PSP promotes immune responses *via* induction of immunoglobulin production and engagement of various pattern-recognition molecules.

### NK Cells

Natural killer cells have a central role in antitumor immune response network. While studies on the immunomodulatory effects of CV extract indicate increased NK cells activity, data on the effects of PSP on NK cells function are scarce. However, similar to other immune cell subsets, many of the changes in cytokine release by PSP can affect the function of NK cells and enhance their activity. For example, TNF-α is a known inducer of NK cell cytotoxic activity (54), particularly in the presence of IL-2 (55). Activated NK cells additionally produce TNF-α upon contact with target cells (56), which, in concert with IFN-γ, can upregulate ICAM-1 on target cancer cells to enhance killing activity (57). TNF-α also plays a crucial role in NK cell recruitment to peritoneal tumor sites (58) and in synergy with IL-1, enhances NK cell proliferative responses to mitogenic cytokines (59, 60). IL-1α and IL-1β enhance production of IFN-γ by NK cells (a classic sign of activation) after mitogen treatment (61, 62). IL-1β also increases a host of activation-related NK markers and favors NK cell maturation from umbilical cord blood precursors (63), while the *in vitro* supplementation of IL-1 can increase the ability of NK cells to bind to target cells (64). NK cytotoxic activity is also enhanced by culture supplementation with IL-12 (65), acting in synergy with IL-2 to enhance IFN-γ production (66). IL-12 alone can increase NK proliferation by induction of IL-2 production (67) and plays a crucial role in NK activation by DCs, wherein its release by the latter enhances NK production of IFN-γ (68). Moreover, IL-12 increases the ability of NK cells to recognize tumor cells expressing CD80 or CD86 (69).

Polysaccharopeptide may also increase NK cytotoxic activity by increasing *FasL* expression *via* IL-18 (70, 71). Similarly, the decrease of TGF-β levels by PSP is beneficial for NK cells, as the cytokine can inhibit NK cytolytic activity and responses to IL-2 stimulation (72, 73), as well as inhibit NK production of IFN-γ, TNF-α, and GM-CSF (74), shown to counter the inhibition of NK cells by monocytes (75). Treatment with M-CSF can also increase murine NK cell activation and responsiveness to IL-2 (76), and its clinical administration mitigates decreased NK counts after chemotherapy (77). The PSP-induced rise in IL-6 production may also enhance NK cell proliferation, TNF-α secretion, and NK adhesive abilities (78). The collective effects of these induced cytokines suggest the possibility of testing PSP in concert with cell therapy to improve the antitumor activity of NK cells.

- *In vitro* and *in vivo* data on the direct effects of PSP on NK cells are scarce.
- The role of PSP on antitumor activity of NK cells remains speculative, opening the possibilities for investigating potential synergistic effects with cellular immunotherapy.

### Immune Modulation by CV Extracts *via* Induction of Superoxide Dismutase (SOD)

Polysaccharopeptide also seems to modulate immunity by regulating the response to oxidative stress, particularly common in cancer patients after myelotoxic regimens. For example, SOD catalyzes the formation of O_2_ or H_2_O_2_ from superoxide radicals (O_2_) and is often downregulated in the tumor microenvironment (79, 80). Injection of healthy mice with CV extract i.p. for 3 days enhanced lymphocyte production of SOD in a dose-dependent manner and partially rescued lymphocyte, splenic, and thymic SOD activities that were reduced by tumor implantation or irradiation of other mice (81). Indeed, the common Japanese strain of CV extract mimics the radical scavenging activity of SOD *in vitro* (82), and its extent is dependent on PSK peptide contents (83). NK cells cultured in the presence of reactive oxygen species showed a gradual and time-dependent decrease in cytolytic activity and an ability to bind target cells, both of which were rescued by PSK (or SOD) treatment. Moreover, i.p. treatment of tumor-bearing rats with PSK or SOD slowed tumor progression, decreased oxidative stress, and restored NK killing and binding activity—effects that were abrogated by an NK-depleting antibody (83). Under oxidative stress, lymphocyte surfaces are thought to become anionic, pointing to a potential mechanism by which SOD reverses this surface charge imbalance and rescues their ability to bind targets (83). Finally, many of the PSP-induced cytokines (TNF-α, IFN-γ, IL-1, and IL-6) have been linked to enhanced SOD activity (84, 85). While SOD is often studied for its anti-inflammatory activity, these data, taken in sum point to the capacity of CV to directly or indirectly scavenge superoxide radicals—a factor that seems to enhance its immunomodulatory abilities.

*Coriolus versicolor* extracts modulate immune cell activity and control tumor progression by reducing superoxide radicals-associated stress in the tumor microenvironment.

## CV in the Clinic: PSP as Adjunct Therapy

While cell culture and animal models are crucial interrogation tools, they are limited—both biologically and technically. Rodent immune systems are intrinsically different in their development, composition, and extent of response to stimuli. Moreover, many of the previously mentioned studies may be unintentionally biased, as they did not report endotoxin testing of the PSP/CV product before its administration (9–12, 16, 23, 25, 33, 38, 39, 47, 53, 81, 86, 87). In addition, while most *in vivo* studies used intraperitoneal (6, 10, 11, 81, 83, 87) or s.c. delivery of PSP (9, 26), they are less relevant physiologically (at least in a clinical context) than models using force-feeding (33), oral delivery (23, 39, 47), or even intragingival injections (25).

The success of PSP in preclinical models has nonetheless prompted many to investigate CV-derived products as potentially adjunctive therapy to standard chemotherapy or radiotherapy regimens for various malignancies. To confirm whether its immunostimulatory effects carry forth from animal models to humans in homeostatic conditions, a double-blind, crossover study recruited healthy volunteers for a 10-month study period. The participants were randomized to receive capsules of PSP (plus another herbal derivative, Danshen) or a placebo, with those receiving PSP showing increased T helper cell counts and percentage, and their PBMCs showing elevated expression of *IL-2R*, as well as increased production of IFN-γ upon stimulation (88). Another randomized trial compared the effects of PSP and amoxicillin on gut microbiome composition and showed effects similar to prebiotic treatment (89), which suggest a benefit on gut/mucosal immunology when delivered orally. Indeed, of the patients positive for oral human papilloma virus treated with CV extract (plus another herbal extract, *ganoderma lucidum*), almost 90% cleared the virus after 2 months of treatment (90). The impact of patient gut microbiota on the success of PSP’s immunomodulatory effects is a surprisingly underinvestigated area that will likely shed light on the mechanisms involved in responses to orally delivered PSP.

It is also of interest to test whether the stimulatory effects of PSP may alleviate the immunoinhibitory environment in cancer patients, particularly after chemotherapy or radiotherapy. In a dose-escalation trial with nine breast cancer patients, CV extract was administered after the completion of radiotherapy and was shown to increase NK cytotoxic function and lymphocyte counts, with CD8^+^ T cells and CD19^+^ B cells increasing dose dependently (91). Similarly, more than 80 previously treated breast cancer patients were given PSP/Danshen capsules for 6 months, leading to increased T-helper and B cell counts and proportions. However, these patients showed significant decreases in serum IL-2R (92), which could indicate that previously reported increases in *IL-2R* gene expression lead to increased surface, and not secretory, levels (88). Increases in leukocyte and neutrophil counts, as well as serum IgG and IgM levels, were observed in non-small cell lung cancer patients randomized to PSP treatment. While no improvements were observed for disease-related parameters, less PSP-treated patients were withdrawn from the study due to disease progression (93). In addition, these patients also showed improvements in their body fat measures, further implicating PSP in affecting the microbiota and/or the mucosal immune system (94, 95). Radiation-induced lymphopenia (particularly T cells) was also alleviated following PSP/Danshen treatment in a randomized, double-blind, placebo-controlled study in nasopharyngeal carcinoma patients (96). Indeed, PSP has been shown to significantly extend the 5-year survival of esophageal cancer patients in a double-blind trial and was reported to relieve pain and enhance immunity in a majority of esophageal, lung, stomach, ovarian, and cervical cancer patients reviewed by Kidd (97). In lung, gastric and esophageal carcinoma, PSP was also associated (to varying degrees) with alleviated symptoms, improved NK activity, increased IL-2 production and CD4 T cell levels, protective effects against radiation-induced lymphopenia, and improved survival rates when combined with radiotherapy. It was also reported to lead to tumor regression in liver carcinoma patients (98). Overall, the clinical application of PSP adjunct therapy for various malignancies suggests disease-related improvement: amelioration of tumor-associated symptoms, reduction in disease progression, and increased survival rates. Rigorous randomized controlled trials are needed to confirm these observations.

## Summary

**Figure 2:**
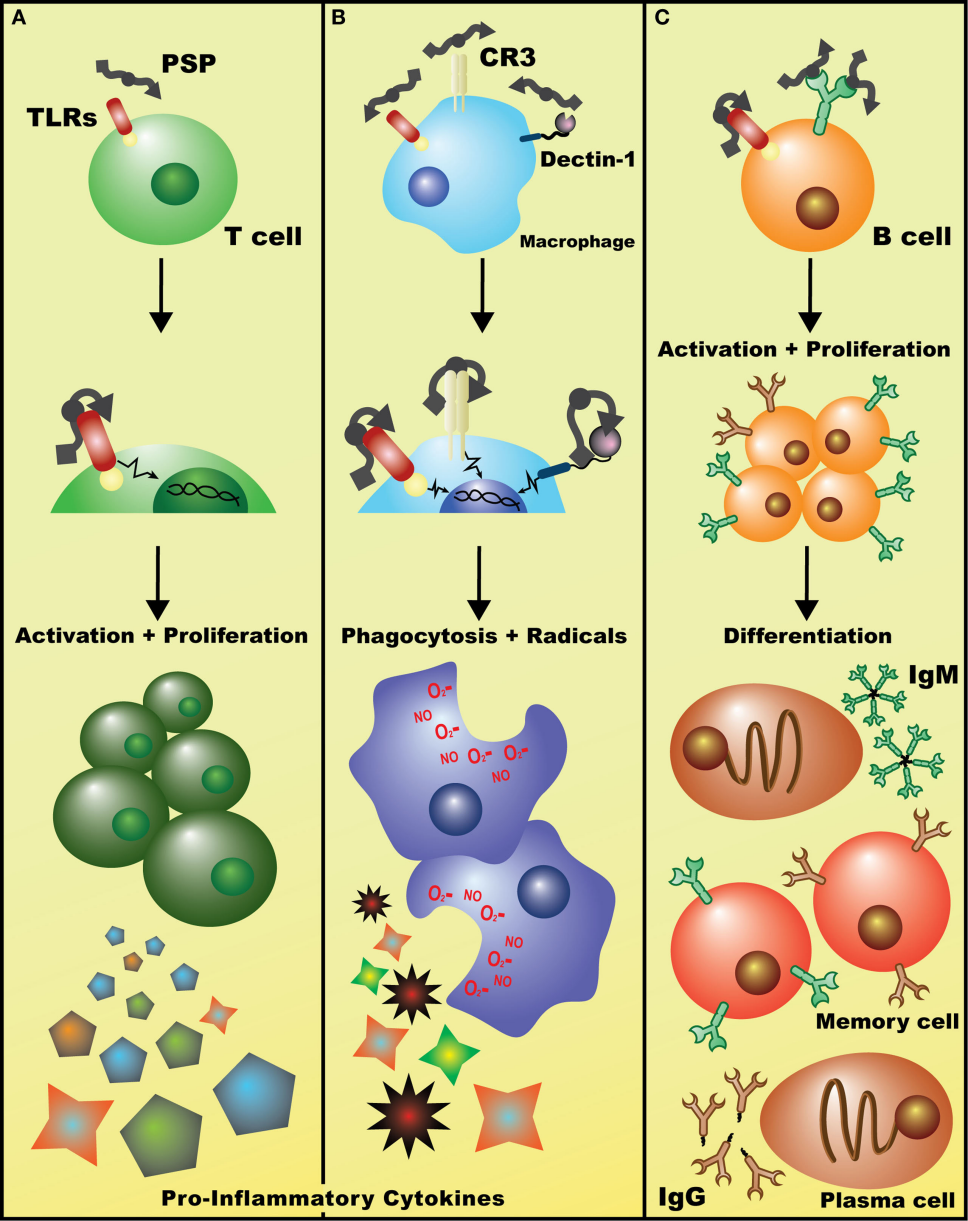
Potential mechanisms of immuostimulatory effects of *Coriolus versicolor*. **(A)** Detection of polysaccharopeptide (PSP) by TLR(s) (and perhaps other receptors) on T lymphocytes initiates signaling cascades, such as the p38 MAPK pathway, leading to enhanced T cell proliferation and the release of largely pro-inflammatory cytokines such as IL-2 and IFN-γ. **(B)** Binding of PSP to any/all of Dectin-1, CR3, or TLRs on macrophages leads to the activation of genetic events that increase phagocytic activity and induces the production of oxidative radicals and cytokines such as tumor necrosis factor-α. **(C)** Recognition of PSP by the BCR leads to B cell activation, clonal proliferation, and eventual differentiation into IgM^+^ or IgG^+^ plasma and memory B cells. Alternatively, PSP may be acting on B cells in a similar fashion to T cells, non-specifically activating them through TLR(s) and leading to a general increase in polyclonal IgM and IgG levels (data not shown).

Preclinical *in vitro* and *in vivo* data suggest that PSP has immunomodulatory (largely immunostimulatory) effects that may be beneficial (summarized in Figure 2; Table 1), particularly when combined with anticancer treatment. The immediate effect of a PSP-mediated cytokine profile on the tumor microenvironment requires further study to determine whether changes operate locally at the tumor site or act systemically to boost the immune system *via* paracrine mechanisms. Most cytokine changes induced by PSP may particularly affect NK cells, acting to enhance cytotoxic activity, trafficking and adhesion to target cells and promote NK cell proliferation. However, it remains to be determined how much of the induced cytokine profile *in vivo* is an indirect effect in response to the tumoricidal activity by PSP, given that it can interrupt the cell cycle, induce apoptosis, and sensitize tumor cells to other chemotherapeutic agents.

**Table 1:** Summary of immuostimulatory effects of *Coriolus versicolor* (CV).

Affected phenotype	*In vitro* (murine)	*In vivo*	*In vitro* (Human)	Clinical
Cytokine profile	IFN-γ: Lym (12) IL-1β: P.Mφ (6) IL-2: Lym (12) IL-12: Lym (12) IL-18: Lym (12) TNFα: P.Mφ (6, 52)	IL-1β: P.Mφ (6) IL-6: Ser (10) M-CSF: P.Mφ (16) Spln (16) TGF-β: CC (9) 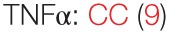 P.Mφ (6, 23), Ser (10, 11)	G-CSF: PBMC (14) GM-CSF: PBMC (14) IFN-γ: PBMC (7) IL-1β: CC (22) IL-1α: PBMC (14) IL-6: CC (22), PBMC (8, 14) IL-12: PBMC (8) TNFα: PBMC (7, 8)	IFN-γ: PBMC (88) IL-2 (98) 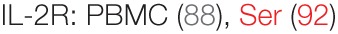
Humoral immunity	Spln IgG1 (38) Spln IgM (38)	Lym IgG (39) Ser IgG (46, 88) Ser IgM (37)	–	LC-Ser IgG (93) LC-Ser IgM (93)
Cell function	Cyto: NK (83)^a^ NO: P.Mφ (52) Phag: Mφ (37), P.Mφ^51^ Prolif: Spln (12), B cell (38) TLR: Spln (38)	Act: Spln (9) Cyto: NK (39) NO: P.Mφ/Mφ (23, 34) PGE_2_: P.Mφ (6) Phag: Mφ (37) Pop #: WBC (46) Neut (46), TC (27, 34) Lym (9), Spln (26) Mφ (27), B cell (27) NK (27, 83)^a^ Prolif: Lym (39) ROS: RBC (83) SO: P.Mφ (23) SOD: Lym (81), Spln (81) TLR: DC (52), TC (52)	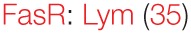 Pop #: Mono (36) Prolif: Lym (35), PBMC (7) TLR: PBMC (8, 14)	Cyto: BrCa-NK (91), NK (98) Pop #: BrCa-Lym (91) BrCa-CD8TC (91) BrCa-CD19 B cell (91, 92) BrCa-Th (92) CD4TC (98) LC-Leuk (93) LC-Neut (93), Lym (98) NPC-TC (96), Th (88)
Tumor growth	–	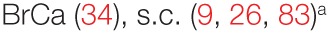	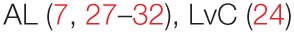	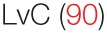
Microbiome	–	–	–	Pre-biotic (89)
Viral clearance	–	–	–	HPV (90)
Survival/slower disease progression	–	–	–	ECa (97, 98) LC (93, 98) GCa (98)
Cancer associated symptoms	–	–	–	BF (93) 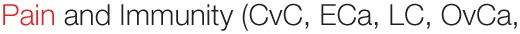 GCa) (97)

*Top row indicates application context of CV product*.

*Act, activation/stimulation; AL, acute leukemia; BrCa, breast cancer; BF, body fat; CC, cancer cells; CvC, cervical cancer; CrC, colorectal cancer; Cyto, cytolytic function; DC, dendritic cells; ECa, esophageal cancer; GCa, stomach or gastric cancer; GI, gastrointestinal cancer; HPV, human papilloma virus; LC, lung cancer; LvC, liver carcinoma; Leuk, leukocytes; Lym, lymphocytes; TGF, transforming growth factor; (P.)Mφ, (peritoneal) macrophages; Mono, monocytes; NK, natural killer; Neut, neutrophil; NO, nitrogen oxide/associated molecules; NPC, nasopharyngeal carcinoma; OvCa, ovarian cancer; PGE_2_, prostoglandin E_2_; Phag, phagocytic activity; Pop #, population count; Prolif, proliferation; RBC, red blood cells; ROS, reactive oxygen species/oxidative stress; s.c., subcutaneous tumor model; Ser, serum; Spln, splenocytes; SO(D), superoxide (dismutase); TC, T cells; Th, T-helper cells; TLR, dependence on/activation of toll-like receptor pathways; WBC, white blood cells*.

*^a^PSK, otherwise PSP or CV extract*.

*Red text, decreased phenotype—otherwise increased*.

Polysaccharopeptide also has notable effects on various other immune subsets. Separate studies on CV extracts or PSP show increased proliferation of rodent splenocytes, T and B cells, NK cells and neutrophils, as well as human PBMCs, lymphocytes. A reduction in lymphocyte Fas receptor levels, and increased monocyte counts can also occur. PSP appears to sensitize tumors to, and alleviate the symptoms of radiation and chemotherapy, but more careful examination of underlying mechanisms is lacking and urgently needed, especially given the increasingly common use of CV extracts as adjunct therapy in some medical communities.

Clinical studies with PSP may be easier to interpret than some of the preclinical data. Although the data may be encouraging, a mechanistic understanding of how PSP induces its immunomodulatory effects remains lacking. Additional data at the cellular and molecular levels after oral administration of PSP are likely to provide impetus for the design and initiation of rigorous prospective trials of adjunct therapy.

## Conclusion

Reports focusing on the mostly positive immunomodulatory effects of CV indicate the need to conduct rigorous studies to identify underlying mechanisms of action. Future work must focus on better defining the specific molecular targets of PSP using transgenic mouse models and loss of function strategies of likely targets. More stringent characterization of the components of PSP to better define the peptide moiety of the molecule is required. Currently, the Protein Data Bank contains only one entry of a protein derived from CV, a polyphenol oxidase (PDB: 1GYC). While the structure of the polysaccharide moiety is informative, identifying the peptide portion of PSP will provide clues to relevant cell surface receptors. The use of established techniques in analytical and medicinal chemistry holds potential for generating more potent drugs based on PSP structure and mechanisms of action and an opportunity to optimize efficacy and mitigate off-target effects.

## Author Contributions

MS and AK determined the scope of the paper. MS conducted the literature review and prepared the manuscript. IR contributed to discussion, revision, and finalization of the manuscript. AK advised MS, supervised the paper’s completion, and revised the manuscript.

## Conflict of Interest Statement

AK has received funding from Purapharm Inc. Hong Kong to investigate the effect of extracts of traditional Chinese herbal medicines, including Yun Zhi, on the cytolytic activity of NK cells. MS and IR declare no conflict of interest.

## References

[1] Pommier Y Leo E Zhang H Marchand C. DNA topoisomerases and their poisoning by anticancer and antibacterial drugs. Chem Biol (2010) 17(5):421–33. 10.1016/j.chembiol.2010.04.012 20534341 PMC7316379

[2] Bjorkholm M. Etoposide and teniposide in the treatment of acute leukemia. Med Oncol Tumor Pharmacother (1990) 7(1):3–10. 2187120 10.1007/BF03000484

[3] Alken S Kelly CM. Benefit risk assessment and update on the use of docetaxel in the management of breast cancer. Cancer Manag Res (2013) 5:357–65. 10.2147/CMAR.S49321 24143122 PMC3798099

[4] Jantan I Ahmad W Bukhari SN. Plant-derived immunomodulators: an insight on their preclinical evaluation and clinical trials. Front Plant Sci (2015) 6:655. 10.3389/fpls.2015.00655 26379683 PMC4548092

[5] Bradley JR. TNF-mediated inflammatory disease. J Pathol (2008) 214(2):149–60. 10.1002/path.2287 18161752

[6] Chan SL Yeung JH Polysaccharide peptides from COV-1 strain of *Coriolus versicolor* induce hyperalgesia via inflammatory mediator release in the mouse. Life Sci (2006) 78(21):2463–70. 10.1016/j.lfs.2005.10.011 16310221

[7] Lee C-L Yang X Wan J The culture duration affects the immunomodulatory and anticancer effect of polysaccharopeptide derived from *Coriolus versicolor*. Enzyme Microb Technol (2006) 38(1–2):14–21. 10.1016/j.enzmictec.2004.10.009

[8] Wang J Dong B Tan Y Yu S Bao YX. A study on the immunomodulation of polysaccharopeptide through the TLR4-TIRAP/MAL-MyD88 signaling pathway in PBMCs from breast cancer patients. Immunopharmacol Immunotoxicol (2013) 35(4):497–504. 10.3109/08923973.2013.805764 23802631

[9] Mao XW Archambeau JO Gridley DS. Immunotherapy with low-dose interleukin-2 and a polysaccharopeptide derived from *Coriolus versicolor*. Cancer Biother Radiopharm (1996) 11(6):393–403. 10.1089/cbr.1996.11.393 10851500

[10] Jedrzejewski T Piotrowski J Kowalczewska M Wrotek S Kozak W. Polysaccharide peptide from *Coriolus versicolor* induces interleukin 6-related extension of endotoxin fever in rats. Int J Hyperthermia (2015) 31(6):626–34. 10.3109/02656736.2015.1046953 26044874

[11] Jedrzejewski T Piotrowski J Wrotek S Kozak W Polysaccharide peptide induces a tumor necrosis factor-alpha-dependent drop of body temperature in rats. J Therm Biol (2014) 44:1–4. 10.1016/j.jtherbio.2014.06.003 25086966

[12] Ho CY Lau CB Kim CF Leung KN Fung KP Tse TF Differential effect of *Coriolus versicolor* (Yunzhi) extract on cytokine production by murine lymphocytes in vitro. Int Immunopharmacol (2004) 4(12):1549–57. 10.1016/j.intimp.2004.07.021 15351324

[13] Sims JE Smith DE. The IL-1 family: regulators of immunity. Nat Rev Immunol (2010) 10(2):89–102. 10.1038/nri2691 20081871

[14] Li W Liu M Lai S Xu C Lu F Xiao X Immunomodulatory effects of polysaccharopeptide (PSP) in human PBMC through regulation of TRAF6/TLR immunosignal-transduction pathways. Immunopharmacol Immunotoxicol (2010) 32(4):576–84. 10.3109/08923971003586876 20131955

[15] Voronov E Dotan S Krelin Y Song X Elkabets M Carmi Y Unique versus redundant functions of IL-1alpha and IL-1beta in the tumor microenvironment. Front Immunol (2013) 4:177 10.3389/fimmu.2013.00177 23847618 PMC3703603

[16] Liu F Fung MC Ooi VE Chang ST. Induction in the mouse of gene expression of immunomodulating cytokines by mushroom polysaccharide-protein complexes. Life Sci (1996) 58(21):1795–803. 10.1016/0024-3205(96)00163-4 8637405

[17] Bogdan C Nathan C. Modulation of macrophage function by transforming growth factor beta, interleukin-4, and interleukin-10. Ann N Y Acad Sci (1993) 685:713–39. 10.1111/j.1749-6632.1993.tb35934.x 8363277

[18] Sanjabi S Zenewicz LA Kamanaka M Flavell RA Anti-inflammatory and pro-inflammatory roles of TGF-beta, IL-10, and IL-22 in immunity and autoimmunity. Curr Opin Pharmacol (2009) 9(4):447–53. 10.1016/j.coph.2009.04.008 19481975 PMC2755239

[19] Scheller J Chalaris A Schmidt-Arras D Rose-John S. The pro- and anti-inflammatory properties of the cytokine interleukin-6. Biochim Biophys Acta (2011) 1813(5):878–88. 10.1016/j.bbamcr.2011.01.034 21296109

[20] Tilg H Trehu E Atkins MB Dinarello CA Mier JW. Interleukin-6 (IL-6) as an anti-inflammatory cytokine: induction of circulating IL-1 receptor antagonist and soluble tumor necrosis factor receptor p55. Blood (1994) 83(1):113–8. 8274730

[21] Lechner MG Liebertz DJ Epstein AL Characterization of cytokine-induced myeloid-derived suppressor cells from normal human peripheral blood mononuclear cells. J Immunol (2010) 185(4):2273–84. 10.4049/jimmunol.1000901 20644162 PMC2923483

[22] Hsieh TC Kunicki J Darzynkiewicz Z Wu JM. Effects of extracts of *Coriolus versicolor* (I’m-Yunity) on cell-cycle progression and expression of interleukins-1 beta,-6, and -8 in promyelocytic HL-60 leukemic cells and mitogenically stimulated and nonstimulated human lymphocytes. J Altern Complement Med (2002) 8(5):591–602. 10.1089/107555302320825101 12470440

[23] Liu WK Ng TB Sze SF Tsui KW. Activation of peritoneal macrophages by polysaccharopeptide from the mushroom, *Coriolus versicolor*. Immunopharmacology (1993) 26(2):139–46. 10.1016/0162-3109(93)90006-C 8282538

[24] Chan SL Yeung JH. Effects of polysaccharide peptide (PSP) from *Coriolus versicolor* on the pharmacokinetics of cyclophosphamide in the rat and cytotoxicity in HepG2 cells. Food Chem Toxicol (2006) 44(5):689–94. 10.1016/j.fct.2005.10.001 16297519

[25] Lin IH Hau DM Chang YH. Restorative effect of *Coriolus versicolor* polysaccharides against gamma-irradiation-induced spleen injury in mice. Zhongguo Yao Li Xue Bao (1996) 17(2):102–4. 9772653

[26] Mao XW Green LM Gridley DS. Evaluation of polysaccharopeptide effects against C6 glioma in combination with radiation. Oncology (2001) 61(3):243–53. 10.1159/000055381 11574781

[27] Hui KP Sit WH Wan JM Induction of S phase cell arrest and caspase activation by polysaccharide peptide isolated from *Coriolus versicolor* enhanced the cell cycle dependent activity and apoptotic cell death of doxorubicin and etoposide, but not cytarabine in HL-60 cells. Oncol Rep (2005)14(1):145–55. 10.3892/or.14.1.145 15944782

[28] Zeng F Hon CC Sit WH Chow KY Hui RK Law IK Molecular characterization of *Coriolus versicolor* PSP-induced apoptosis in human promyelotic leukemic HL-60 cells using cDNA microarray. Int J Oncol (2005) 27(2):513–23. 10.3892/ijo.27.2.513 16010435

[29] Hsieh TC Wu P Park S Wu JM Induction of cell cycle changes and modulation of apoptogenic/anti-apoptotic and extracellular signaling regulatory protein expression by water extracts of I’m-Yunity (PSP). BMC Complement Altern Med (2006) 6:30 10.1186/1472-6882-6-30 16965632 PMC1574346

[30] Wan JM Sit WH Yang X Jiang P Wong LL. Polysaccharopeptides derived from *Coriolus versicolor* potentiate the S-phase specific cytotoxicity of camptothecin (CPT) on human leukemia HL-60 cells. Chin Med (2010) 5:16. 10.1186/1749-8546-5-16 20423495 PMC2874562

[31] Yang X Sit WH Chan DK Wan JM. The cell death process of the anticancer agent polysaccharide-peptide (PSP) in human promyelocytic leukemic HL-60 cells. Oncol Rep (2005) 13(6):1201–10. 10.3892/or.13.6.1201 15870943

[32] Li XY. Immunomodulating components from Chinese medicines. Pharm Biol (2000) 38(Suppl 1):33–40. 10.1076/phbi.38.6.33.5961 23531136

[33] Wang HX Ng TB Liu WK Ooi VE Chang ST. Polysaccharide-peptide complexes from the cultured mycelia of the mushroom *Coriolus versicolor* and their culture medium activate mouse lymphocytes and macrophages. Int J Biochem Cell Biol (1996) 28(5):601–7. 10.1016/1357-2725(95)00157-3 8697105

[34] Luo KW Yue GG Ko CH Lee JK Gao S Li LF In vivo and in vitro anti-tumor and anti-metastasis effects of *Coriolus versicolor* aqueous extract on mouse mammary 4T1 carcinoma. Phytomedicine (2014) 21(8–9):1078–87. 10.1016/j.phymed.2014.04.020 24856767

[35] Lee CL Jiang P Sit WH Yang X Wan JM. Regulatory properties of polysaccharopeptide derived from *Coriolus versicolor* and its combined effect with ciclosporin on the homeostasis of human lymphocytes. J Pharm Pharmacol (2010) 62(8):1028–36. 10.1211/jpp.62.08.0009 20663037

[36] Sekhon BK Sze DM Chan WK Fan K Li GQ Moore DE PSP activates monocytes in resting human peripheral blood mononuclear cells: immunomodulatory implications for cancer treatment. Food Chem (2013) 138(4):2201–9. 10.1016/j.foodchem.2012.11.009 23497877

[37] Sze DM Chan GC. Supplements for immune enhancement in hematologic malignancies. Hematology Am Soc Hematol Educ Program (2009) 2009:313–9. 10.1182/asheducation-2009.1.313 20008216

[38] Yang SF Zhuang TF Si YM Qi KY Zhao J *Coriolus versicolor* mushroom polysaccharides exert immunoregulatory effects on mouse B cells via membrane Ig and TLR-4 to activate the MAPK and NF-kappaB signaling pathways. Mol Immunol (2015) 64(1):144–51. 10.1016/j.molimm.2014.11.007 25480394

[39] Qian ZM Xu MF Tang PL. Polysaccharide peptide (PSP) restores immunosuppression induced by cyclophosphamide in rats. Am J Chin Med (1997) 25(1):27–35. 10.1142/S0192415X97000068 9166995

[40] Ricciotti E FitzGerald GA. Prostaglandins and inflammation. Arterioscler Thromb Vasc Biol (2011) 31(5):986–1000. 10.1161/ATVBAHA.110.207449 21508345 PMC3081099

[41] Krause P Bruckner M Uermosi C Singer E Groettrup M Legler DF. Prostaglandin E(2) enhances T-cell proliferation by inducing the costimulatory molecules OX40L, CD70, and 4-1BBL on dendritic cells. Blood (2009) 113(11):2451–60. 10.1182/blood-2008-05-157123 19029446

[42] Yao C Sakata D Esaki Y Li Y Matsuoka T Kuroiwa K Prostaglandin E2-EP4 signaling promotes immune inflammation through Th1 cell differentiation and Th17 cell expansion. Nat Med (2009) 15(6):633–40. 10.1038/nm.1968 19465928

[43] Legler DF Krause P Scandella E Singer E Groettrup M. Prostaglandin E2 is generally required for human dendritic cell migration and exerts its effect via EP2 and EP4 receptors. J Immunol (2006) 176(2):966–73. 10.4049/jimmunol.176.2.966 16393982

[44] Holt DM Ma X Kundu N Collin PD Fulton AM. Modulation of host natural killer cell functions in breast cancer via prostaglandin E2 receptors EP2 and EP4. J Immunother (2012) 35(2):179–88. 10.1097/CJI.0b013e318247a5e9 22306906 PMC3721982

[45] Jonuleit H Kuhn U Muller G Steinbrink K Paragnik L Schmitt E Pro-inflammatory cytokines and prostaglandins induce maturation of potent immunostimulatory dendritic cells under fetal calf serum-free conditions. Eur J Immunol (1997) 27(12):3135–42. 10.1002/eji.1830271209 9464798

[46] Degli-Esposti MA Smyth MJ. Close encounters of different kinds: dendritic cells and NK cells take centre stage. Nat Rev Immunol (2005) 5(2):112–24. 10.1038/nri1549 15688039

[47] Sekhon BK Roubin RH Li Y Devi PB Nammi S Fan K Evaluation of selected immunomodulatory glycoproteins as an adjunct to cancer immunotherapy. PLoS One (2016) 11(1):e0146881. 10.1371/journal.pone.0146881 26799072 PMC4723152

[48] Barsanti L Passarelli V Evangelista V Frassanito AM Gualtieri P Chemistry, physico-chemistry and applications linked to biological activities of beta-glucans. Nat Prod Rep (2011) 28(3):457–66. 10.1039/c0np00018c 21240441

[49] Wan JM Sit WH Louie JC. Polysaccharopeptide enhances the anticancer activity of doxorubicin and etoposide on human breast cancer cells ZR-75-30. Int J Oncol (2008) 32(3):689–99. 10.3892/ijo.32.3.689 18292947

[50] Hong F Yan J Baran JT Allendorf DJ Hansen RD Ostroff GR Mechanism by which orally administered beta-1,3-glucans enhance the tumoricidal activity of antitumor monoclonal antibodies in murine tumor models. J Immunol (2004) 173(2):797–806. 10.4049/jimmunol.173.2.797 15240666

[51] Taylor PR Brown GD Reid DM Willment JA Martinez-Pomares L Gordon S The beta-glucan receptor, dectin-1, is predominantly expressed on the surface of cells of the monocyte/macrophage and neutrophil lineages. J Immunol (2002) 169(7):3876–82. 10.4049/jimmunol.169.7.3876 12244185

[52] Kang SC Koo HJ Park S Lim JD Kim YJ Kim T Effects of beta-glucans from *Coriolus versicolor* on macrophage phagocytosis are related to the Akt and CK2/Ikaros. Int J Biol Macromol (2013) 57:9–16. 10.1016/j.ijbiomac.2013.03.017 23500440

[53] Lu H Yang Y Gad E Wenner CA Chang A Larson ER Polysaccharide krestin is a novel TLR2 agonist that mediates inhibition of tumor growth via stimulation of CD8 T cells and NK cells. Clin Cancer Res (2011) 17(1):67–76. 10.1158/1078-0432.CCR-10-1763 21068144 PMC3017241

[54] Voth R Rossol S Gallati H Pracht I Laubenstein HP Hess G In vivo and in vitro induction of natural killer cells by cloned human tumor necrosis factor. Cancer Immunol Immunother (1988) 27(2):128–32. 10.1007/BF00200016 2458181 PMC11038699

[55] Ayroldi E Sorci G Cannarile L Riccardi C. Effect of recombinant murine tumor necrosis factor on the generation of natural killer cells in bone marrow cultures. Nat Immun (1992) 11(2):92–104. 1498522

[56] Fauriat C Long EO Ljunggren HG Bryceson YT. Regulation of human NK-cell cytokine and chemokine production by target cell recognition. Blood (2010) 115(11):2167–76. 10.1182/blood-2009-08-238469 19965656 PMC2844017

[57] Wang R Jaw JJ Stutzman NC Zou Z Sun PD Natural killer cell-produced IFN-gamma and TNF-alpha induce target cell cytolysis through up-regulation of ICAM-1. J Leukoc Biol (2012) 91(2):299–309. 10.1189/jlb.0611308 22045868 PMC3290424

[58] Smyth MJ Kelly JM Baxter AG Korner H Sedgwick JD. An essential role for tumor necrosis factor in natural killer cell-mediated tumor rejection in the peritoneum. J Exp Med (1998) 188(9):1611–9. 10.1084/jem.188.9.1611 9802973 PMC2212521

[59] Robertson MJ Cameron C Lazo S Cochran KJ Voss SD Ritz J. Costimulation of human natural killer cell proliferation: role of accessory cytokines and cell contact-dependent signals. Nat Immun (1996) 15(5):213–26. 9390270

[60] Robertson MJ Manley TJ Donahue C Levine H Ritz J. Costimulatory signals are required for optimal proliferation of human natural killer cells. J Immunol (1993) 150(5):1705–14. 7679691

[61] Cooper MA Fehniger TA Ponnappan A Mehta V Wewers MD Caligiuri MA.Interleukin-1beta costimulates interferon-gamma production by human natural killer cells. Eur J Immunol (2001) 31(3):792–801. 10.1002/1521-4141(200103)31:3<792::AID-IMMU792>3.0.CO;2-U 11241284

[62] Hunter CA Timans J Pisacane P Menon S Cai G Walker W Comparison of the effects of interleukin-1 alpha, interleukin-1 beta and interferon-gamma-inducing factor on the production of interferon-gamma by natural killer. Eur J Immunol (1997) 27(11):2787–92. 10.1002/eji.1830271107 9394800

[63] Ambrosini P Loiacono F Conte R Moretta L Vitale C Mingari MC IL-1beta inhibits ILC3 while favoring NK-cell maturation of umbilical cord blood CD34(+) precursors. Eur J Immunol (2015) 45(7):2061–71. 10.1002/eji.201445326 25847448

[64] Herman J Dinarello CA Kew MC Rabson AR. The role of interleukin 1 (IL 1) in tumor-NK cell interactions: correction of defective NK cell activity in cancer patients by treating target cells with IL 1. J Immunol (1985) 135(4):2882–6. 2993420

[65] Naume B Gately M Espevik T A comparative study of IL-12 (cytotoxic lymphocyte maturation factor)-, IL-2-, and IL-7-induced effects on immunomagnetically purified CD56+ NK cells. J Immunol (1992) 148(8):2429–36. 1373169

[66] Chan SH Perussia B Gupta JW Kobayashi M Pospisil M Young HA Induction of interferon gamma production by natural killer cell stimulatory factor: characterization of the responder cells and synergy with other inducers. J Exp Med (1991) 173(4):869–79. 10.1084/jem.173.4.869 1672545 PMC2190821

[67] Perussia B Chan SH D’Andrea A Tsuji K Santoli D Pospisil M Natural killer (NK) cell stimulatory factor or IL-12 has differential effects on the proliferation of TCR-alpha beta+, TCR-gamma delta+ T lymphocytes, and NK cells. J Immunol (1992) 149(11):3495–502. 1358972

[68] Borg C Jalil A Laderach D Maruyama K Wakasugi H Charrier S NK cell activation by dendritic cells (DCs) requires the formation of a synapse leading to IL-12 polarization in DCs. Blood (2004) 104(10):3267–75. 10.1182/blood-2004-01-0380 15242871

[69] Geldhof AB Moser M Lespagnard L Thielemans K De Baetselier P. Interleukin-12-activated natural killer cells recognize B7 costimulatory molecules on tumor cells and autologous dendritic cells. Blood (1998) 91(1):196–206. 9414285

[70] Hashimoto W Osaki T Okamura H Robbins PD Kurimoto M Nagata S Differential antitumor effects of administration of recombinant IL-18 or recombinant IL-12 are mediated primarily by Fas-Fas ligand- and perforin-induced tumor apoptosis, respectively. J Immunol (1999) 163(2):583–9. 10395644

[71] Takeda K Tsutsui H Yoshimoto T Adachi O Yoshida N Kishimoto T Defective NK cell activity and Th1 response in IL-18-deficient mice. Immunity (1998) 8(3):383–90. 10.1016/S1074-7613(00)80543-9 9529155

[72] Ortaldo JR Mason AT O’Shea JJ Smyth MJ Falk LA Kennedy IC Mechanistic studies of transforming growth factor-beta inhibition of IL-2-dependent activation of CD3- large granular lymphocyte functions. Regulation of IL-2R beta (p75) signal transduction. J Immunol (1991) 146(11):3791–8. 1851793

[73] Rook AH Kehrl JH Wakefield LM Roberts AB Sporn MB Burlington DB Effects of transforming growth factor beta on the functions of natural killer cells: depressed cytolytic activity and blunting of interferon responsiveness. J Immunol (1986) 136(10):3916–20. 2871107

[74] Bellone G Aste-Amezaga M Trinchieri G Rodeck U. Regulation of NK cell functions by TGF-beta 1. J Immunol (1995) 155(3):1066–73. 7636180

[75] van den Bosch G Preijers F Vreugdenhil A Hendriks J Maas F De Witte T. Granulocyte-macrophage colony-stimulating factor (GM-CSF) counteracts the inhibiting effect of monocytes on natural killer (NK) cells. Clin Exp Immunol (1995) 101(3):515–20. 10.1111/j.1365-2249.1995.tb03143.x 7664499 PMC1553231

[76] Misawa E Sakurai T Yamada M Hayasawa H Motoyoshi K. Effects of macrophage colony-stimulating factor and interleukin-2 administration on NK1.1(+) cells in mice. Int J Immunopharmacol (2000) 22(11):967–77. 10.1016/S0192-0561(00)00061-8 11090705

[77] Hidaka T Akada S Teranishi A Morikawa H Sato S Yoshida Y Mirimostim (macrophage colony-stimulating factor; M-CSF) improves chemotherapy-induced impaired natural killer cell activity, Th1/Th2 balance, and granulocyte function. Cancer Sci (2003) 94(9):814–20. 10.1111/j.1349-7006.2003.tb01524.x 12967481 PMC11160279

[78] Rabinowich H Sedlmayr P Herberman RB Whiteside TL. Response of human NK cells to IL-6 alterations of the cell surface phenotype, adhesion to fibronectin and laminin, and tumor necrosis factor-alpha/beta secretion. J Immunol (1993) 150(11):4844–55. 8496590

[79] Oberley LW Buettner GR. Role of superoxide dismutase in cancer: a review. Cancer Res (1979) 39(4):1141–9. 217531

[80] Parascandolo A Rappa F Cappello F Kim J Cantu DA Chen H Extracellular superoxide dismutase expression in papillary thyroid cancer mesenchymal stem/stromal cells modulates cancer cell growth and migration. Sci Rep (2017) 7:41416. 10.1038/srep41416 28216675 PMC5316948

[81] Wei WS Tan JQ Guo F Ghen HS Zhou ZY Zhang ZH Effects of *Coriolus versicolor* polysaccharides on superoxide dismutase activities in mice. Zhongguo Yao Li Xue Bao (1996) 17(2):174–8. 9772673

[82] Kariya K Nakamura K Nomoto K Matama S Saigenji K. Mimicking of superoxide dismutase activity by protein-bound polysaccharide of *Coriolus versicolor* QUEL, and oxidative stress relief for cancer patients. Mol Biother (1992) 4(1):40–6. 1627273

[83] Nakamura K Matsunaga K Susceptibility of natural killer (NK) cells to reactive oxygen species (ROS) and their restoration by the mimics of superoxide dismutase (SOD). Cancer Biother Radiopharm (1998) 13(4):275–90. 10.1089/cbr.1998.13.275 10850363

[84] Harris CA Derbin KS Hunte-McDonough B Krauss MR Chen KT Smith DM Manganese superoxide dismutase is induced by IFN-gamma in multiple cell types. Synergistic induction by IFN-gamma and tumor necrosis factor or IL-1. J Immunol (1991) 147(1):149–54. 1904900

[85] Tsan MF White JE Del Vecchio PJ Shaffer JB. IL-6 enhances TNF-alpha- and IL-1-induced increase of Mn superoxide dismutase mRNA and O_2_ tolerance. Am J Physiol (1992) 263(1 Pt 1):L22–6. 1636726 10.1152/ajplung.1992.263.1.L22

[86] Klir JJ McClellan JL Kozak W Szelenyi Z Wong GH Kluger MJ Systemic but not central administration of tumor necrosis factor-alpha attenuates LPS-induced fever in rats. Am J Physiol (1995) 268(2 Pt 2):R480–6. 7864244 10.1152/ajpregu.1995.268.2.R480

[87] Yang MM Chen Z Kwok JS. The anti-tumor effect of a small polypeptide from *Coriolus versicolor* (SPCV). Am J Chin Med (1992) 20(3–4):221–32. 10.1142/S0192415X92000230 1471606

[88] Wong CK Tse PS Wong EL Leung PC Fung KP Lam CW Immunomodulatory effects of yun zhi and danshen capsules in health subjects – a randomized, double-blind, placebo-controlled, crossover study. Int Immunopharmacol (2004) 4(2):201–11. 10.1016/j.intimp.2003.12.003 14996412

[89] Pallav K Dowd SE Villafuerte J Yang X Kabbani T Hansen J Effects of polysaccharopeptide from *Trametes versicolor* and amoxicillin on the gut microbiome of healthy volunteers: a randomized clinical trial. Gut Microbes (2014) 5(4):458–67. 10.4161/gmic.29558 25006989

[90] Donatini B. Control of oral human papillomavirus (HPV) by medicinal mushrooms, *Trametes versicolor* and *Ganoderma lucidum*: a preliminary clinical trial. Int J Med Mushrooms (2014) 16(5):497–8. 10.1615/IntJMedMushrooms.v16.i5.80 25271984

[91] Torkelson CJ Sweet E Martzen MR Sasagawa M Wenner CA Gay J Phase 1 clinical trial of *Trametes versicolor* in women with breast cancer. ISRN Oncol (2012) 2012:251632. 10.5402/2012/251632 22701186 PMC3369477

[92] Wong CK Bao YX Wong EL Leung PC Fung KP Lam CW. Immunomodulatory activities of Yunzhi and Danshen in post-treatment breast cancer patients. Am J Chin Med (2005) 33(3):381–95. 10.1142/S0192415X05002990 16047556

[93] Tsang KW Lam CL Yan C Mak JC Ooi GC Ho JC *Coriolus versicolor* polysaccharide peptide slows progression of advanced non-small cell lung cancer. Respir Med (2003) 97(6):618–24. 10.1053/rmed.2003.1490 12814145

[94] Brown K DeCoffe D Molcan E Gibson DL. Diet-induced dysbiosis of the intestinal microbiota and the effects on immunity and disease. Nutrients (2012) 4(8):1095–119. 10.3390/nu4081095 23016134 PMC3448089

[95] Stenman LK Burcelin R Lahtinen S. Establishing a causal link between gut microbes, body weight gain and glucose metabolism in humans – towards treatment with probiotics. Benef Microbes (2016) 7(1):11–22. 10.3920/BM2015.0069 26565087

[96] Bao YX Wong CK Leung SF Chan AT Li PW Wong EL Clinical studies of immunomodulatory activities of Yunzhi-Danshen in patients with nasopharyngeal carcinoma. J Altern Complement Med (2006) 12(8):771–6. 10.1089/acm.2006.12.771 17034283

[97] Kidd PM. The use of mushroom glucans and proteoglycans in cancer treatment. Altern Med Rev (2000) 5(1):4–27. 10696116

[98] Ng TB A review of research on the protein-bound polysaccharide (polysaccharopeptide, PSP) from the mushroom *Coriolus versicolor* (Basidiomycetes: Polyporaceae). Gen Pharmacol (1998) 30(1):1–4. 10.1016/S0306-3623(97)00076-1 9457474

